# Gender pay gaps and inequity at science publishers

**DOI:** 10.1371/journal.pgph.0004673

**Published:** 2025-06-10

**Authors:** Jocalyn Clark, Elizabeth Zuccala

**Affiliations:** 1 International Editor, The BMJ, London, United Kingdom; 2 Senior Deputy Medical Editor, Medical Journal of Australia, Sydney, Australia; PLOS: Public Library of Science, UNITED STATES OF AMERICA

*Jocalyn Clark and Elizabeth Zuccala show that, in contrast to commitments to and recognition for equity, diversity, inclusion and gender equality efforts, leading science publishers have maintained large and persistent gender pay gaps favouring men since 2018, despite promises to close the gender gap.*

The world’s largest science publishers wield enormous influence – publishing research and commentary that informs practice and policy globally and advancing the values of health, sustainability, and collaboration. Over the past decade, science publishers have embraced a role and responsibility in promoting equity, diversity, and inclusion (EDI) in research [1], and in particular have, since 2018 amidst the #metoo and #timesup era, taken a leading position to overcome gender inequality, including in their own publishing practices [2–5],

A key dimension of gender equality is valuing women’s work and ensuring their economic empowerment. A key indicator of gender equality in workplaces is transparency and equality of pay. Since 2017, the United Kingdom (UK) has mandated organisations employing more than 250 people to publicly report their annual gender pay gap [https://gender-pay-gap.service.gov.uk/]. Eight years of data, across more than 10,000 companies in 2024, are now available to assess the extent to which gaps exist in how leading science publishers pay the women and men on their staff, and to review progress since 2018 when we first wrote in *The Lancet* about the inequities of gender gaps in pay [6]. Of course, leading publishers don’t just support research – they profit handsomely from it via the massive US$32.1 billion annual science publishing market [7]. Therefore, their employment practices deserve scrutiny especially in light of stated commitments to equality.

## What is the gender pay gap?

The gender pay gap refers to the difference in median hourly pay of all men and women in a company’s workforce. Gender differences in pay do not necessarily mean unequal pay between men and women for the same job, which is illegal in the UK. But any gaps will expose the predominance of men in higher ranking, more highly remunerated positions, may be suggestive of discrimination in access to higher paying jobs, and are an indication of how women are being undervalued by their employers. Gaps will also indicate potential deficiencies in the fairness of workplace environments and policies including those to do with recruitment, training, part-time employment, people with caring responsibilities, managing the impacts of career breaks, and other forms of support for career advancement.

**Fig 1:**
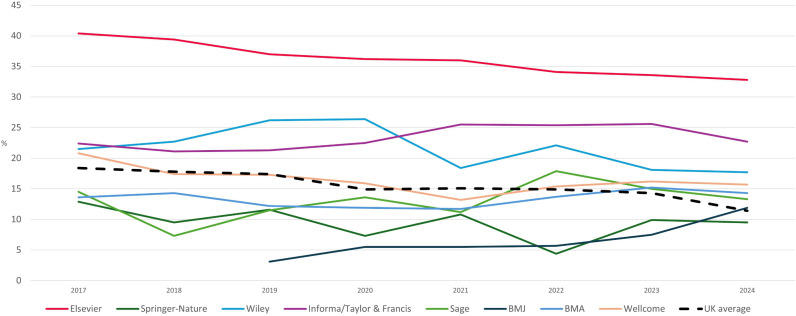
Median gender pay gap over time, UK science publishers and organisations. Source: https://gender-pay-gap.service.gov.uk/.

We collected data from 2017 to 2024 for the five largest science publishers in the UK, which publish some of the highest impact health and science journals in the world including *Nature, Science*, *Cell*, and *The Lancet.* For additional comparison we collected data for the publisher and owner of a leading UK-based medical journal, *The* *BMJ*, and the largest UK-based science funder, Wellcome (Table 1, Fig 1).

**Table 1:** Median pay gap of UK-based science organisations over time.

	2017	2018	2019	2020	2021	2022	2023	2024
*UK average*	*18.4*	*17.8*	*17.4*	*14.9*	*15.1*	*14.9*	*14.3*	*11.4*
Elsevier	40.4	39.4	37	36.2	36	34.1	33.6	32.8
Springer-Nature	12.9	9.5	11.6	7.3	10.8	4.4	9.9	9.5
Wiley	21.5	22.7	26.2	26.4	18.4	22.1	18.1	17.7
Informa/ Taylor & Francis	22.4	21.1	21.3	22.5	25.5	25.4	25.6	22.7
Sage	14.5	7.3	11.5	13.6	11.2	17.9	15.0	13.3
BMJ^*^	n/a	n/a	3.1	5.5	5.5	5.7	7.5	11.9
BMA	12.6	14.3	12.2	11.9	11.7	13.7	15.2	14.3
Wellcome	20.8	17.4	17.3	15.9	13.2	15.4	16.2	15.7

Median gap between women and men in hourly pay of UK-based organisations publicly reporting at https://gender-pay-gap.service.gov.uk/. Mandatory reporting is due on April 5 of the subsequent year.

* personal communication.

n/a = not available

Every science publisher pays men more than women. In 2024, the lowest median pay gap favouring men was 9.5% (Springer-Nature), followed by Sage (13.3%), Wiley (17.7%), and Informa (formerly Taylor & Francis) (22.7%).

Elsevier remains an outlier in the magnitude of its gender pay gap and in the lack of progress. Eight years ago Elsevier stood out among publishers, with a median pay gap in 2017 of 40.4% in favour of men over women in its UK business. The UK average that year was 18.4% and we called Elsevier’s gap – double the national average – unacceptable [6]. The company’s leadership promised change [6].

Elsevier’s median pay gap for 2024 is 32.8%, maintaining its position as worst performer among peers over all eight years of mandatory reporting, and tracking only a slight improvement of seven percentage points over time. In fact, the ratio of Elsevier’s pay gap to the UK average has worsened – from 2.2 times in 2017 and 2.4 in 2020 and 2021 to now 2.9 times the UK average in 2024.

Peer companies such as Springer-Nature and Wiley have decreased their pay gaps by 26% (to 9.5%) and by 18% (to 17.7%), respectively, and the BMJ has never reported a gender pay gap over 12%. Wellcome reports for 2024 a pay gap of 15.7%, down from a high of 20.8% for 2017 – which their director described at the time as “uncomfortable” and committed to changing by installing a gender-balanced executive leadership team, introducing gender bias training for staff, and creating new recruitment processes and pay structures [8, 9].

The pay differences in our sample of publishers compare unfavourably to related sectors. Global Health 50/50 reported a decline from 12.7% to 10.8% between 2017 and 2022 in the median pay gaps of 43 UK-based global health organisations [10]. In the global health care sector overall, the International Labour Organization and WHO reported a gender pay gap of 24% favouring men in 2022 [11].

## What are the solutions?

Basic best practices to decrease gender pay gaps range from EDI recruitment practices that use inclusive language and expand talent pools, to pay audits, to mentoring schemes and transparency in salary and promotions [12].

Broader strategies recognise the extent to which the gender pay gap is driven by occupational segregation whereby professions and roles dominated by women are undervalued compared with those of men – a product of discrimination against women’s labour [13]. The publishing industry is feminising while maintaining a predominance of male senior leaders and higher earners; unless corrected, the current gender pay gap risks internal occupational segregation whereby women experience what has been called a “wage penalty” [13].

To be clear, the problem here is not a lack of qualified or motivated women to occupy and excel in senior editorial and publishing roles, which often require advanced scientific or medical training and expertise. Equal numbers of women have entered science and medical school for decades. Men advance in careers where women experience barriers because there is unequal access to recruitment and promotion, occupational segregation, gender stereotypes against women, and the motherhood penalty. Therefore, it is not a problem in women to be fixed but the responsibility of publishers to provide conducive environments for women’s career advancement, free of bias that limits their access to upward mobility and higher pay.

## Making excuses instead of change

Gender pay gaps at science and health publishers – especially persistent gaps – defy all manner of commitments to EDI and gender equality. The world’s three largest science publishers, Elsevier, Springer-Nature and Wiley, attract considerable accolades for their corporate social responsibility, sustainability, and equality efforts. Commitments to diversity and inclusion are prominently displayed on their journals’ pages and in global marketing and branding efforts, and in the case of Elsevier, a high-profile Inclusion and Diversity Advisory Board of pre-eminent experts is said to provide guidance to the company and address inequality [14]. Accountability seems strikingly lacking. As we argued previously [6], “it is one thing to cite “diversity” and “inclusion” on a corporate website, but quite another to design and implement strategies that will make a real difference.”

After eight years of mandatory reporting (which tracks but doesn’t fine or sanction) and no parity in pay, there needs to be stronger demands and measures for all leading scientific organisations and publishers to account for and address this problem. Change at the outlier, Elsevier, which is growing in dominance [15], seems especially overdue. Transparency is necessary for exposing inequities, but on its own is insufficient for ensuring accountability for change. Although major science publishers wield substantial power, they do so in large part because they rely on the trust, cooperation, expertise, and labour of a large global scientific community that they purport to serve. Principles of EDI and gender equality are fundamental to doing good science – they should be bedrocks of publishing that science too.
